# Niobium K-Edge X-ray Absorption Spectroscopy of Doped TiO_2_ Produced from Ilmenite Digested in Hydrochloric Acid

**DOI:** 10.1021/acsomega.2c02676

**Published:** 2022-08-04

**Authors:** Richard G. Haverkamp, Peter Kappen, Katie H. Sizeland, Kia S. Wallwork

**Affiliations:** † School of Engineering and Advanced Technology, Massey University, Private Bag 11222, Palmerston North 4442, New Zealand; ‡ Australian Synchrotron, ANSTO, Clayton 3168, Victoria, Australia

## Abstract

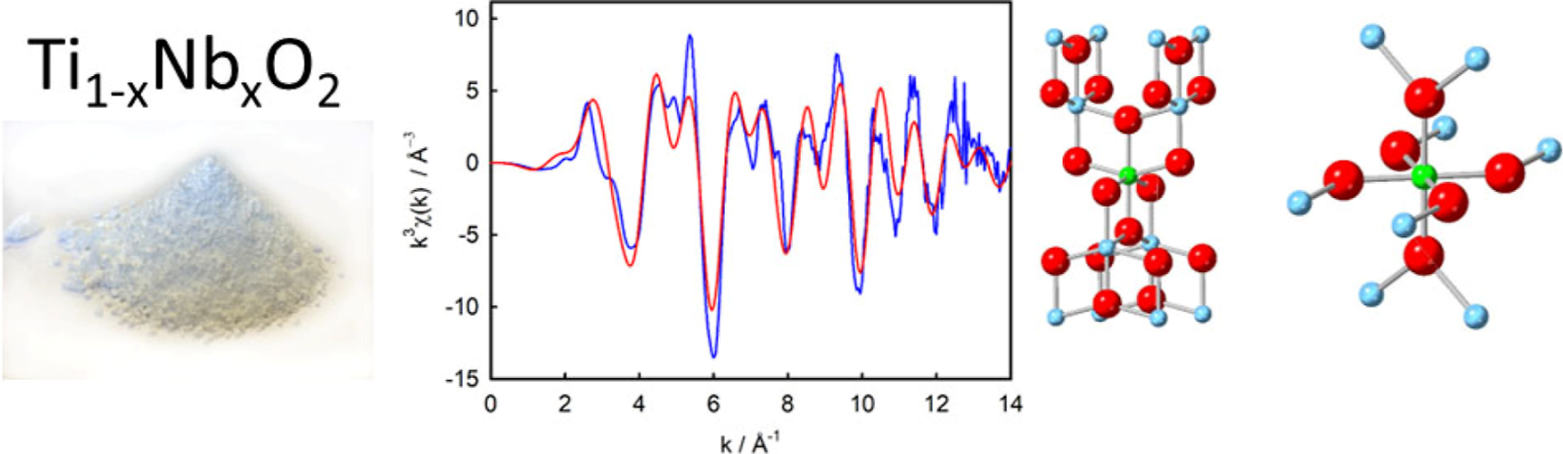

Niobium doping of TiO_2_ creates a conductive material with many new energy applications. When TiO_2_ is precipitated from HCl solutions containing minor Nb, the Nb in solution is quantitatively deposited with the TiO_2_. Here, we investigate the structure of Nb doped in anatase and rutile produced from ilmenite digested in hydrochloric acid. Nb K-edge X-ray absorption near edge structure (XANES) and extended X-ray absorption fine structure (EXAFS) are used to characterize the environment of 0.08 atom % Nb doped in TiO_2_. XANES shows clear structural differences between Nb-doped anatase and rutile. EXAFS for Nb demonstrates that Nb occupies a Ti site in TiO_2_ with no near neighbors of Nb. Hydrolysis of Ti and Nb from acid solution, followed by calcination, leads to a well dispersed doped material, with no segregation of Nb. Production of Nb-doped TiO_2_ by this method may be able to supply future demand for large quantities of the material and in energy applications where a low cost of production, from readily available natural resources, would be highly desirable.

## 1 Introduction

The dominant use of TiO_2_ is for the white pigment.^1^ However, many non-pigment applications of TiO_2_ have been developed or are being considered. One important feature of TiO_2_ that enables new applications is the ability to modify this normally insulating material to become an electrical conductor. One way to produce this conductivity is by doping of TiO_2_ with niobium. If these applications are to become economical, a means to produce Nb-doped TiO_2_ on an industrial scale is required.

A proposed production method for TiO_2_ from the relatively abundant titanium containing ore, ilmenite, is the digestion in HCl^2^ and subsequent precipitation of a titanium oxide hydrate directly from solution,^3^ followed by calcination. In this process, Nb naturally present in the ilmenite dissolves and is quantitatively precipitated with the TiO_2_. Either rutile or anatase forms can be made directly or anatase formed and converted into rutile by calcination.

Nb-doped TiO_2_ can act similar to a transparent metal which could be used in the place of indium-tin-oxide (Sn-doped In_2_O_3_), which is widely used in flat panel displays, touch panels, and light-emitting devices.^4^ Other uses for Nb-doped TiO_2_ include photovoltaics^5^ and dye-sensitized solar cells.^6−8^ It also finds application in photocatalysis^9^ including for the production of H_2_,^10^ photoelectrochemical water splitting,^11^ and photocatalytic CO_2_ reduction.^12^ Other catalytic applications are for catalyst supports,^13^ for example, for the oxygen reduction reaction^14^ and H_2_ production,^15^ or for dimensionally stable anodes for the chlorine evolution reaction,^16^ and electrochemical destruction of “forever chemicals”.^17^ Nb-doped TiO_2_ has potential application in batteries and supercapacitors^18^ including lithium-ion batteries^19,20^ and Na-ion batteries.^21^ Other potentials uses are for CO sensing^22^ and thermoelectric power production.^23^

These many possible uses of Nb-doped TiO_2_, many of which are in new energy developments, suggest that there could be future demand for large quantities of the material and in applications where a low cost of production would be required. Therefore, a process to produce the doped material on a large scale from readily available natural resources is highly desirable.

For doping of TiO_2_ to display modified electronic properties, it may be necessary for the dopant to be distributed throughout the material. Therefore, any method for the bulk production of doped TiO_2_ should also evaluate the nature of the incorporation of the dopant.

In this work, the nature of the incorporation of Nb into TiO_2_ produced from hydrolysis of HCl solutions of Barrytown, New Zealand ilmenite is investigated. X-ray absorption near edge structure (XANES) and extended X-ray absorption fine structure (EXAFS), supported by X-ray diffraction (XRD), are used to characterize the local environment of Nb in both anatase and rutile phases of TiO_2_. The purpose is to determine whether Nb occupies Ti sites without significantly modifying the TiO_2_ structure or if Nb is intermixed as a distinct oxide phase.

## 2 Experimental Section

### 2.1 Preparation of TiO_2_

Placer ilmenite from Barrytown, New Zealand, containing 0.05% Nb_2_O_5_, was digested in 35 wt % hydrochloric acid and precipitated as TiO_2_ hydrate with either rutile or anatase structure, as described in more detail elsewhere.^3^ Rutile hydrate is the natural product from the hydrolysis of HCl solutions, while the anatase hydrate was obtained by the addition of H_3_PO_4_ in the seeding stage of the hydrolysis (equivalent to 0.35% P_2_O_5_ in the final TiO_2_ product). The hydrate, after the addition of KCl to the level of 0.3% K_2_O, was calcined at 925 °C for 1 h, thus yielding the TiO_2_ material used in this study.

### 2.2 Elemental Analysis

The elemental composition of the titanium dioxide hydrates,^3^ prior to calcination, were determined by Spectrachem Analytical Services, Lower Hutt, New Zealand. The analyses were performed on a Siemens SRS303AS wavelength-dispersive X-ray fluorescence spectrometer. Pressed powder samples were used with the Siemens “Spectraplus” semi-quantitative multi-element analysis.

### 2.3 X-ray Diffraction

XRD was performed at the bending magnet powder diffraction beamline at the Australian Synchrotron. This beamline uses a Mythen II silicon microstrip detector with an intrinsic angular resolution of 0.004°.^24^ For the experiments, the wavelength was set at λ = 0.58959 (1) Å (*E* = 21 keV), and the vertical beam size was 0.4–0.5 mm at the sample. Samples were packed in 0.3 mm quartz capillaries, with 0.01 mm wall thickness (W. Müller, Schönwalde). The wavelength was determined accurately through Rietveld analysis of the diffraction pattern from LaB_6_.

### 2.4 X-ray Absorption Spectroscopy

X-ray absorption spectroscopy (XAS) was performed at the wiggler XAS beamline at the Australian Synchrotron. Samples were finely ground with a mortar and pestle and pressed into pellets. Spectra across the Nb K-edge (*E*_0_ = 18,985.6 eV^25^) were recorded in the fluorescence mode with a 100-element detector (Canberra). The samples were held in a He-cooled cryostat (*T* < 20 K). Energy steps of 10 eV pre-edge and 0.35 eV across the edge (1 s/step) were used. In the EXAFS range, *k*-steps of 0.035 Å^–1^ (up to 5 s/step) were used. The energy scale was calibrated by simultaneously measuring a Nb foil placed between the two downstream ion chambers. The photon flux at the sample was around 10^10^ photons s^–1^. No signs of radiation damage were detected from repeat scans, permitting multiple scans to be summed in order to improve signal-to-noise. Reference standards were Nb foil as well as 0.02% NbO_2_ (Aldrich) and Nb_2_O_5_ (Aldrich) both diluted to 0.02% in boric acid and loaded into 1 mm thick sample holders. The beam size at the sample was about 1.5 × 0.4 mm (*H* × *V*).

XANES and EXAFS data were processed using the freeware package Athena/Artemis,^26^ with scattering paths provided through FEFF6.^27^

## 3 Results and Discussion

### 3.1 X-ray Diffraction

**Figure 1:**
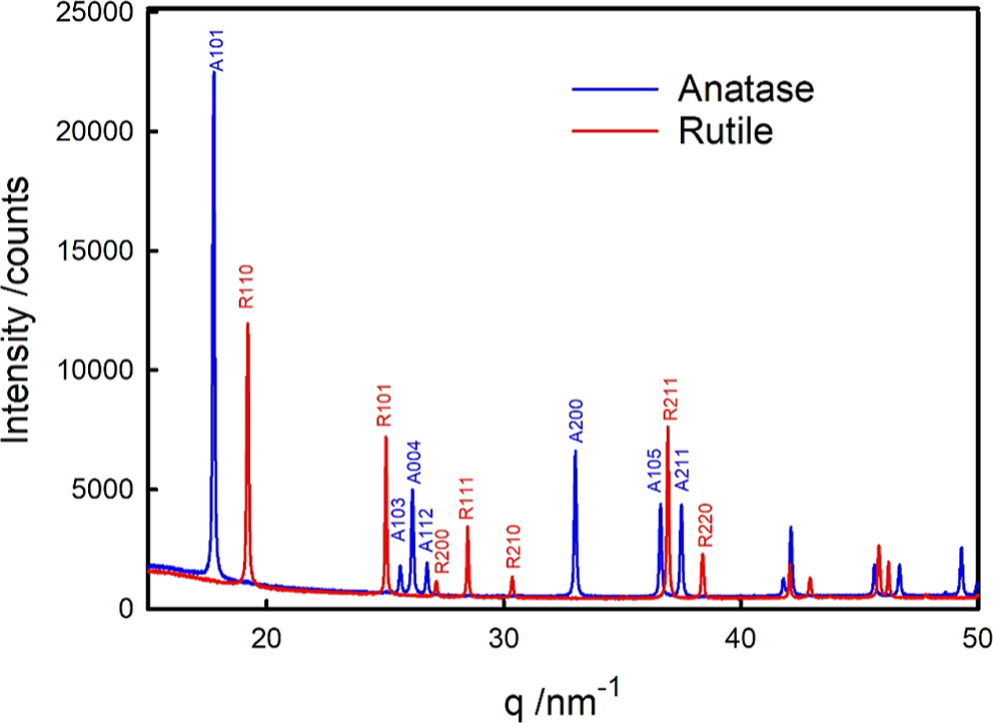
X-ray diffraction patterns of Nb-doped anatase (blue) and Nb-doped rutile (red) with the peaks identified by their Miller indices (and indicated A for an anatase diffraction, R for a rutile diffraction).

The two samples of TiO_2_ prepared for this study are shown to be highly crystalline anatase or rutile (Figure 1) with no detectable admixture of the two phases in either sample and no other phases present.

### 3.2 Elemental Composition

The TiO_2_ hydrates that were the basis for the anatase and rutile materials used in this work both contained Nb at the level of 0.13% “Nb_2_O_5_” (see Table 1, taken from ref (3)); this oxide represents the conventional method to convey elemental concentrations and does not mean the oxide must be in this form. The reported concentration corresponds to 0.08 atom % Nb of the Ti + Nb. These also contain phosphorous, a portion of which comes from inclusions in the ilmenite ore^28^ and with additional phosphorous added to produce anatase instead of the naturally formed rutile. Si, Al, Ca, and K (except the K added later as a calcination flux) are likely to be present as discrete finely divided gangue material, from inclusions in the ilmenite ore, that passed through filtration steps and became incorporated in the hydrolysis product and subsequently in the calcined material. This work did not specifically ascertain the form of the Si, Al, Ca, and K, but as the solubility of these materials is low in hydrochloric acid used for the ore digestion. Small inclusions of minerals such as garnet and feldspar are typically present in ilmenite, thus posing a likely route to the inclusion of these elements in the rutile and anatase precipitates.

**Table 1:** Elemental Composition of the Two TiO_2_ Materials Prior to Calcination^a^

element as oxide	anatase wt %	rutile wt %
TiO_2_	94.3	95.8
P_2_O_5_	0.82	0.49
Nb_2_O_5_	0.13	0.13
Ta_2_O_5_	0.009	0.009
Cl	4.4	3.1
FeO	0.015	0.044
SiO_2_	0.19	0.28
Al_2_O_3_	0.07	0.03
CaO	0.01	0.02
K_2_O	0.006	0.01

a Reprinted with permission from Haverkamp, R. G.; Wallwork, K.; Waterland, M.; Gu, Q.; Kimpton, J. A. *Ind. Eng. Chem. Res*. **2022,***61* (19), 6333–634. Copyright 2022 American Chemical Society.^3^

### 3.3 X-ray Absorption Near Edge Structure

**Figure 2:**
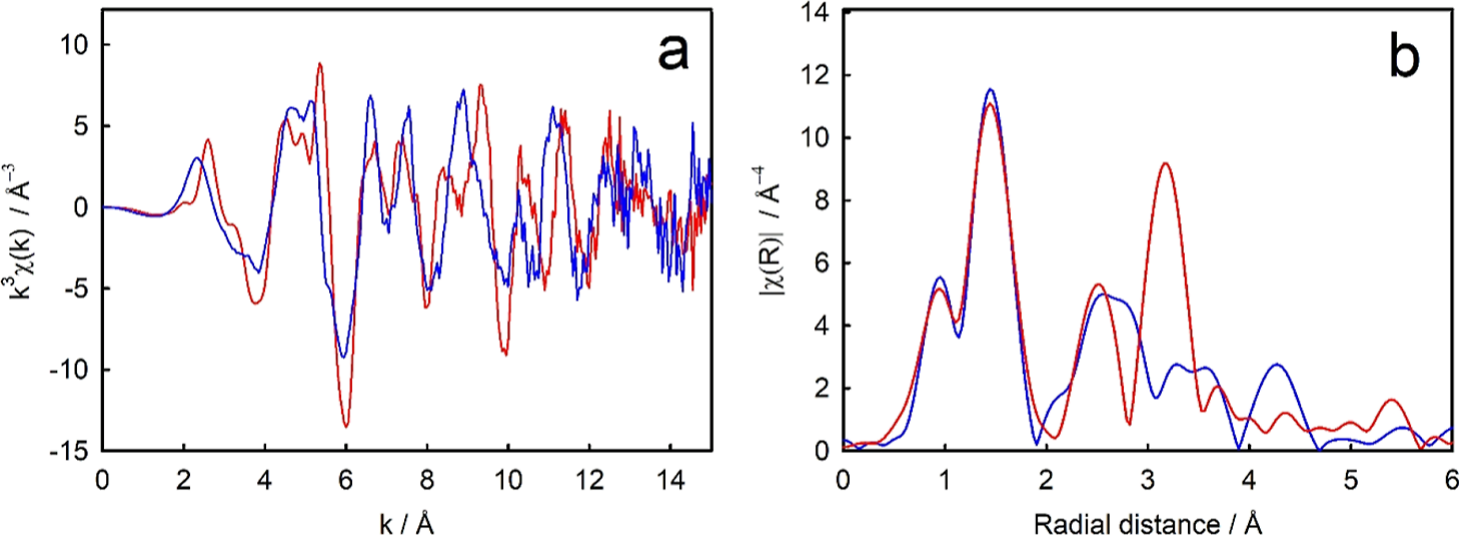
Nb K-edge XANES of Nb-doped TiO_2_ (anatase and rutile) and Nb oxide standards (offset by +0.3).

The primary purpose of this work is to determine the nature of the Nb present in the TiO_2_ of the two different structures. XANES can be used to compare materials of interest with reference materials, primarily providing chemical information. Here, we compare the Nb-doped anatase with the Nb-doped rutile and with two niobium oxide reference compounds. There are clear differences in the Nb K-edge XANES between the Nb-rutile and the Nb-anatase forms (Figure 2). The edge energy is the same in both Nb-doped anatase and rutile (at ∼18,988 eV). However, there are significant differences above the absorption edge, both in the whiteline and further into the XANES region. The reference compounds (Figure 2) contain Nb in NbO_2_ with oxidation state 4+ and Nb_2_O_5_ with oxidation state 5+. The Nb_2_O_5_ has an edge energy of 19,000.2 eV which is similar to that of the Nb-doped TiO_2_ materials and also contains a pre-edge feature similar to that present in those doped materials. The NbO_2_ has a lower edge energy of 18,997.7 eV than Nb_2_O_5_, reflecting the lower oxidation state of NbO_2_. The edge for the Nb metal at 18985.6 eV lies at a lower energy than these oxides.^25^ From the XANES, it is therefore apparent that Nb in the doped anatase and rutile has a different chemical/structural environment in each of the two forms. A published XANES study of 7 atom % Nb doping in anatase TiO_2_ presents a similar spectrum to the 0.08 atom % Nb-doped TiO_2_ here for the anatase form.^9^ XANES of 1.5% Nb in anatase TiO_2_ has also recently been published, where ab initio finite difference method near edge structure using the density functional theory simulation was used to model the spectrum providing good agreement between the data and the model of Nb in anatase.^29^

### 3.4 Extended X-ray Absorption Fine Structure

**Figure 3:**
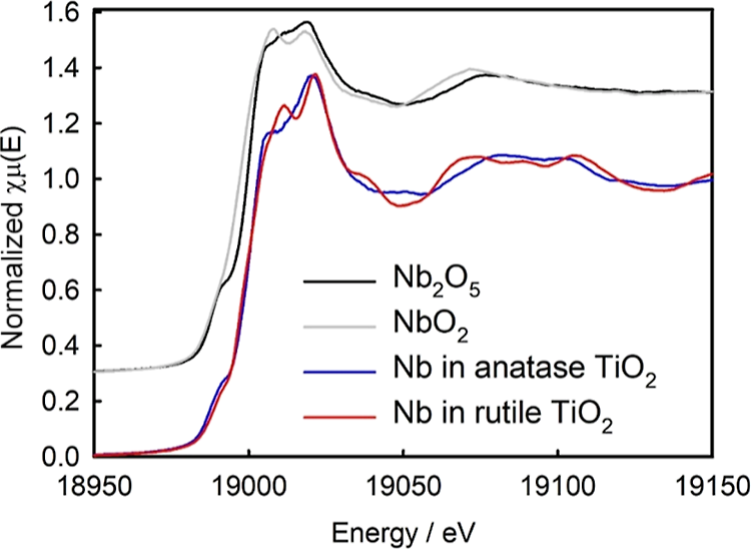
Comparison of recorded spectra for Nb K-edge EXAFS of Nb-doped anatase (blue) and Nb-doped rutile (red) TiO_2_. (a) *k*^3^, (b) Fourier transform.

The Nb K-edge EXAFS of the doped anatase and rutile produced markedly different spectra. The Fourier transforms of these spectra, plotted as radial distance versus the magnitude of the Fourier transform (Figure 3), show that the structural environments of Nb in these two materials are quite different.

The EXAFS was therefore analyzed by solving the EXAFS equation for crystal structures that might be present and comparing these with the experimental data using the following procedure.

TiO_2_ structures were obtained from the Crystallography Open Database^30^ for anatase and rutile, as well as a wide range of niobium oxide structures. For the oxides, structure models were loaded into *Artemis* as is. For Nb-doped TiO_2_ structures, the *Atoms* routine in *Artemis* was run with Nb set as the absorber in place of Ti; scattering paths and phases were then calculated using FEFF. Due to the low concentration of Nb, it was assumed that Nb was isolated on the scale of the attenuation length of electrons in the structure. This assumption was tested against experimental data by fitting. Anatase and rutile structures (with Nb substituted) were evaluated, as were a wide variety of Nb_2_O_5_ and NbO_2_ structures. None of the niobium oxide structures gave good fits, and these attempted fits are provided in the Supporting Information.

**Figure 4:**
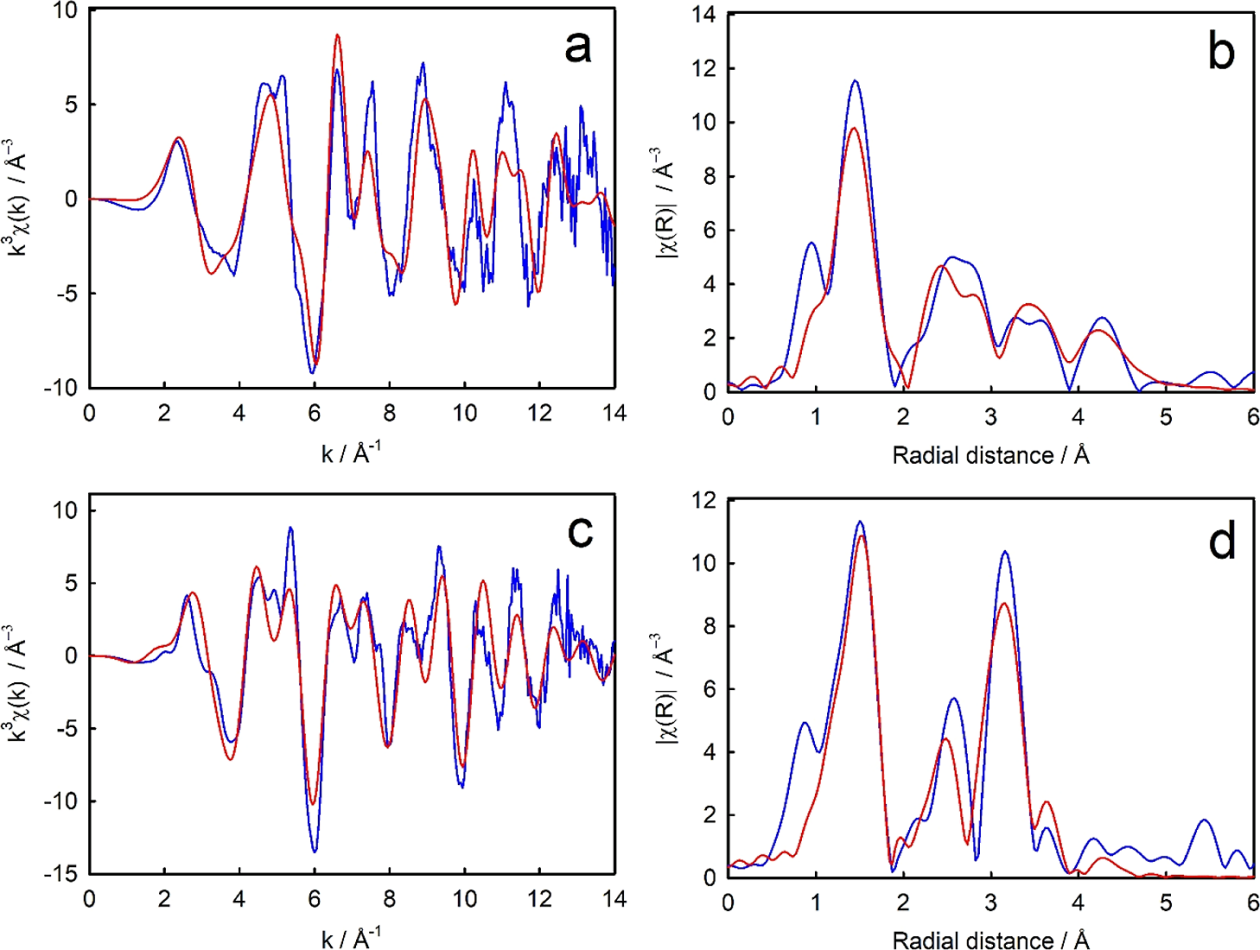
Nb K-edge EXAFS structure fits to data for Nb substituted in a Ti site in *k*-space and *R*-space; (a,b) anatase; and (c,d) rutile. Recorded spectra are shown in blue and *Artemis* fits to the data for the best fit crystal structures are shown in red.

Fitting of the structures to the EXAFS data was performed in real space (*R*), with the quality of fit parameters shown in Table 2. Fairly good fits were obtained, with a fit to a Nb substituted for Ti in anatase and a Nb substituted for Ti in rutile matching well to the experimental data for the Nb-doped anatase and rutile samples, respectively (Figure 4). These unconstrained fits gave *S*_0_^2^ close to 1 in both cases (1.05 ± 0.23 and 1.06 ± 0.14) which provides good confidence that the fitted model in each case is appropriate.

**Table 2:** EXAFS Structure Fitting Conditions and Quality of Fit Parameters

sample	structure fitted (CIF file)	data range used, *k* (Å^–1^)	fitting range in *R* (Å)	reduced χ^2^	*R* factor	*S*_0_^2^ (error)	Δ*R*	σ^2^ (Debye–Waller)
Nb-doped anatase	1010942 anatase^31^	2–11.5	1.01–6	99	0.19	1.05 (0.22)	0.068	0.0058
Nb-doped rutile	9004141 rutile^32^	2–14	1.01–6	35	0.16	1.06 (0.14)	0.048	0.0067

**Figure 5:**
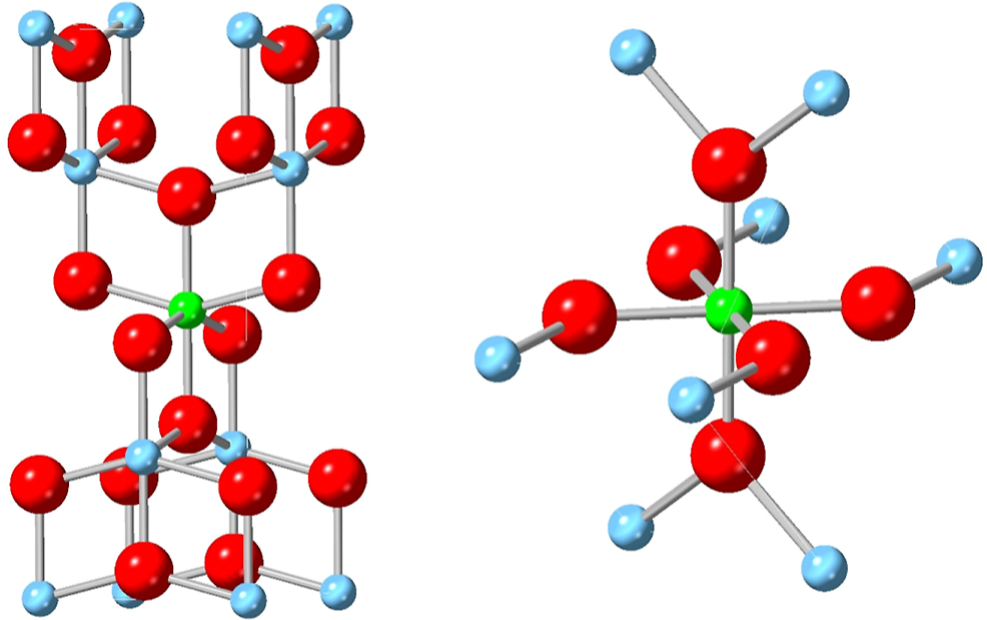
Crystal structures with Nb in anatase (left) and Nb in rutile (right). Green, Nb; blue, Ti; red, O. Generated using CrystalDiffract, CrystalMaker Software Ltd, Oxford, England (www.crystalmaker.com).

The EXAFS fit reveals that Nb is substituted into Ti sites of the crystal structure adopted in each case, anatase or rutile, as represented in the structures shown in Figure 5. The Nb is well dispersed and does not appreciably interact with other Nb atoms in the structure, and it is not present as niobium oxide clusters within TiO_2_ of clusters separate to TiO_2_, consistent with the XANES data discussed above. The EXAFS results also indicate that it is unlikely that Nb forms dimers as has been postulated in some Nb-doped TiO_2_.^33^ In other studies, anatase thin films were prepared from a composite TiNb target by reactive magnetron sputtering giving a level of 1.5% Nb doped in TiO_2_ anatase.^29^ The authors conclude that “the local environment of Nb atoms in the film is close to that of Ti atoms in the anatase phase [...]. This suggests that the substitution of Ti by Nb ions occurs in the film without a strong influence on the TiO_2_ matrix”. Here, we come to a similar conclusion for these bulk materials formed by hydrolysis and with both anatase and rutile where the Nb takes Ti sites in either structure without appreciably modifying the structure. However, it is noted that in other work with TiO_2_ doped to 2.9 atom % Nb or higher, there was a phase change from anatase to rutile on calcination the Nb was segregated, leading to the formation of NbO nanoclusters on the surface of the TiO_2_ rutile nanoparticles even before the phase change took place.^34^ In the work described here, we see no segregation of Nb in the calcined material, with the data supporting highly dispersed Nb substituting for Ti in TiO_2_ structures. This is a positive consideration for energy applications of this material, as discussed further below (Section 3.7).

### 3.5 Industrial Production of Nb-Doped TiO_2_

If the many potential applications of Nb-doped TiO_2_ are to be realized, a low-cost and scalable method to produce such a material is highly desirable. Ilmenite is the main primary source of TiO_2_ and sometimes also contains niobium. The Barrytown, New Zealand ilmenite used here is from a large placer deposit and has an average concentration of 0.05% Nb_2_O_5_ (350 ppm Nb) in the bulk ilmenite with 200–800 ppm Nb in individual ilmenite grains.^28^ It is one potential source for the large-scale production of Nb-doped TiO_2_ materials and has been shown to be amenable to this hydrothermal method both being highly soluble in HCl^2^ and readily precipitated^3^ and calcined to form either anatase or rutile. This process is similar to the “sulfate process” for the large-scale production of pigment grade TiO_2_ which uses sulfuric acid rather than hydrochloric acid.

Other ilmenite deposits have also been reported to contain Nb at useful levels. A West Australian sand deposit contains 1000 ppm Nb on average with up to 3500 ppm Nb in some mineral grains.^35^ Ilmenite from Richard’s Bay, South Africa, contains 460 ppm Nb.^36^ An ilmenite sand deposit, at Walikale, North Kivu, Democratic Republic of Congo, contains 157 ppm Nb on average with 40–341 ppm Nb in individual grains.^37^ An ilmenite deposit in Kuru town, Jos South, Plateau State, Nigeria may have up to 4.4% Nb.^38^ Some of these deposits may be suitable for this process; however, only some ilmenites are readily soluble in hydrochloric acid and other elements present in these ores may also contribute either favorably or unfavorably to the product formed.

The Nb doping level could be increased by the addition of extra Nb, either by concentration from the digestion solutions, adding a soluble Nb ore to the digestion process, or by the addition of a suitable Nb salt to the digestion liquor prior to hydrolysis. The XANES and EXAFS presented here have demonstrated that the Nb is incorporated into the lattice of TiO_2_ by this preparation method.

Production of Nb-doped TiO_2_ has been proposed by sol–gel synthesis, for example, for anatase beads with 0.1–10 atom % Nb from starting material of titanium(IV) isopropoxide with 1-hexadecylamine as a structure-directing agent for use in Li-ion batteries.^20^ A similar sol–gel synthesis of TiO_2_ doped with about 10% Nb from titanium tetrabutyl titanate with 1-hexadecylamine and polydimethylsiloxane as structure determining agents was prepared for use in electrorheological fluids.^39^ A sol–gel preparation followed by spark plasma sintering with repeated oxidation and reduction is another method proposed for the preparation of Nb-doped TiO_2_ powders.^33^ Another possible synthesis route is by grinding precursors of TiNb_2_O_7_ and TiO_2_ in a mechanochemical synthesis^15^ which results in a mixture with a gradient in Nb concentration with more Nb on the surface. However, for large-scale production, preparation by the hydrolysis of aqueous acid solutions to form TiO_2_ is well established on an industrial scale and therefore readily adaptable to produce Nb-doped material.

### 3.6 Other Effects of Nb on TiO_2_ Production

The action of doping TiO_2_ with Nb at hydrolysis to produce a material suitable for the many proposed applications may lead to changes to other properties of the material. The anatase to rutile phase change may be retarded by increased Nb doping^34,40−44^ which may be desirable in some circumstances (when anatase in the desired end product) but undesirable in other circumstances (e.g., more flux may be required to produce rutile from anatase at a suitably small particle size). Final product color may be influenced by the presence of Nb, giving a blue tint. However, this color change is often desirable for pigments and can counteract a yellow tint produced by iron or some other impurities.^9,43,45,46^

### 3.7 Why the Placement of Nb is Important for the Electrical and Optical Properties

It has been shown here that Nb doped into TiO_2_ substitutes in the Ti site for either anatase or rutile and can therefore be fully dispersed within the TiO_2_ material. It is important that Nb does this, rather than forming discrete clusters, in order for the electronic, optical, and photocatalytic properties to be realized.

The electrical conductivity of Nb-doped TiO_2_ depends on both the level of Nb doping with the conductivity increasing approximately linearly with Nb content over the range 0.003–0.03 atom % Nb.^47^ The electrical conductivity is also dependent on the number of oxygen vacancies. The Nb(V) substituting for Ti(IV) requires a charge balance which is met by oxygen deficiency. However, oxygen deficiency can also result from the reducing conditions in the treatment of the Nb-doped TiO_2_. Under mildly reducing conditions (in a study of 0.65 atom % Nb in TiO_2_), an n-type semiconductor is formed, whereas under strongly reducing conditions metallic charge transport is developed.^48^ A density functional theory calculation (screened exchange hybrid functional method) showed that shallow conduction bands should be present in Nb-doped anatase TiO_2_, but deep conduction bands in rutile TiO_2_.^49^ The calculations suggested that Nb donors are compensated by interstitial oxygen anions except at low oxygen partial pressures and low O pressures prevent O interstitials being formed rather than create extra O vacancies. If too much O is removed, the material is no longer transparent as a thin film.^49^

We therefore might expect the Nb-doped rutile form produced in the work presented here to be less electrically conductive than the Nb-doped anatase form, when compared in similar oxygen partial pressure environments. We have shown that the Nb is placed in the Ti sites rather than as dimers or a discrete phase and this enables the electronic properties of the doped material to be realized.

## 4 Conclusions

Niobium-doped TiO_2_, at a level of 0.08 atom % Nb, was produced by the hydrolysis of liquor from the digestion of ilmenite in hydrochloric acid as either anatase or rutile and calcined to form Nb-doped anatase or rutile. XANES of these doped materials showed that Nb in the doped anatase and rutile has a different chemical/structural environment in each of the two forms and the Nb is not in the form of a previously known Nb oxide structure. An EXAFS analysis revealed that Nb is substituted into Ti sites of the crystal structure adopted in each case, anatase or rutile, and that Nb is well dispersed and does not appreciably interact with other Nb atoms in the structure and is not present as niobium oxide clusters within TiO_2_. Segregation of Nb did not occur on calcination. This placement of Nb in Ti sites, well dispersed, is important in order for the electronic, optical, and photocatalytic properties to be realized. Because this method produces Nb substituted into Ti sites and because it is analogous to current industrial scale TiO_2_ production methods, it may be a suitable low cost method of producing Nb-doped material to realize the many potential applications of Nb-doped TiO_2_, especially if Nb levels are boosted by the addition of Nb to the hydrolysis solution..

## Supporting Information Available

EXAFS attempted fits of structures that are not a good match to the data (PDF)
