# Supplementary material for: Niobium K-Edge X-ray Absorption Spectroscopy of Doped TiO2 Produced from Ilmenite Digested in Hydrochloric Acid
Source: ACS Omega. 2022 Aug 4;7(32):28258–64. doi: 10.1021/acsomega.2c02676 (PMC9386810; doi:10.1021/acsomega.2c02676)
Supplement: Supplementary file 1 — ao2c02676_si_001.pdf [file ao2c02676_si_001.pdf]

Supporting information for

Niobium K-Edge X-ray Absorption Spectroscopy of Doped TiO2 Produced from Ilmenite Digested in Hydrochloric Acid

Richard G. Haverkamp,†, Peter Kappen‡, Katie H. Sizeland‡, Kia S. Wallwork‡*

†School of Engineering and Advanced Technology, Massey University, Private Bag 11222, Palmerston North 4442, New Zealand; ‡Australian Synchrotron, ANSTO, Clayton 3168, VIC, Australia

*Email: r.haverkamp@massey.ac.nz

Many structures were compared with the data and deemed to be a poor fit and therefore not the structure of the materials analyzed. This judgement was made based on the R-factor (smaller is better), the energy loss factor S_0^2 (<1 and close to 1 is better), and a visual match of the fitted plots in k-space and in R (the shape matches). There are many possibilities for fitting, including constraining S_0^2 to 1, changing the range of k-space used for the data, fitting to k or R, if fitting to k then to k, k^2 or k^3, choosing only some paths, for example. Shown below are representative examples of fits, in this case using paths up to 5 Å, fitted to R. Many other possible variations on these fits are possible, but for none of these was a reasonable match obtained. In some cases, the R-factor was fairly low but a visual inspection of the fit clearly shows a poor fit. This document shows just the rejected structures. The successful fits are shown in the main paper.

Table S1. Goodness of fit to rejected structures

Structure (CIF, compound, space group)	Fit to data for Nb-doped anatase (R-factor, S_0^2)	Fit to data for Nb-doped rutile (R-factor, S_0^2)

9008684 NbO F m -3 m	0.65, 0.70	0.54, 0.93
9008782 NbO P m -3 m	0.63, 1.01	0.51, 1.5
1520791 NbO_2 I 41/a :2	0.45, 1.5	0.56, 1.9
1534156 Nb_2O_5 C1 2/m 1	0.67, 2.5	0.60, 2.2
1528723 Nb_2O_5 I 4/m m m	0.74, 4.1	0.70, 4.7
1534619 Nb_4O_5 P 42/n m c :2	0.38, 2.2	0.69, 3.3

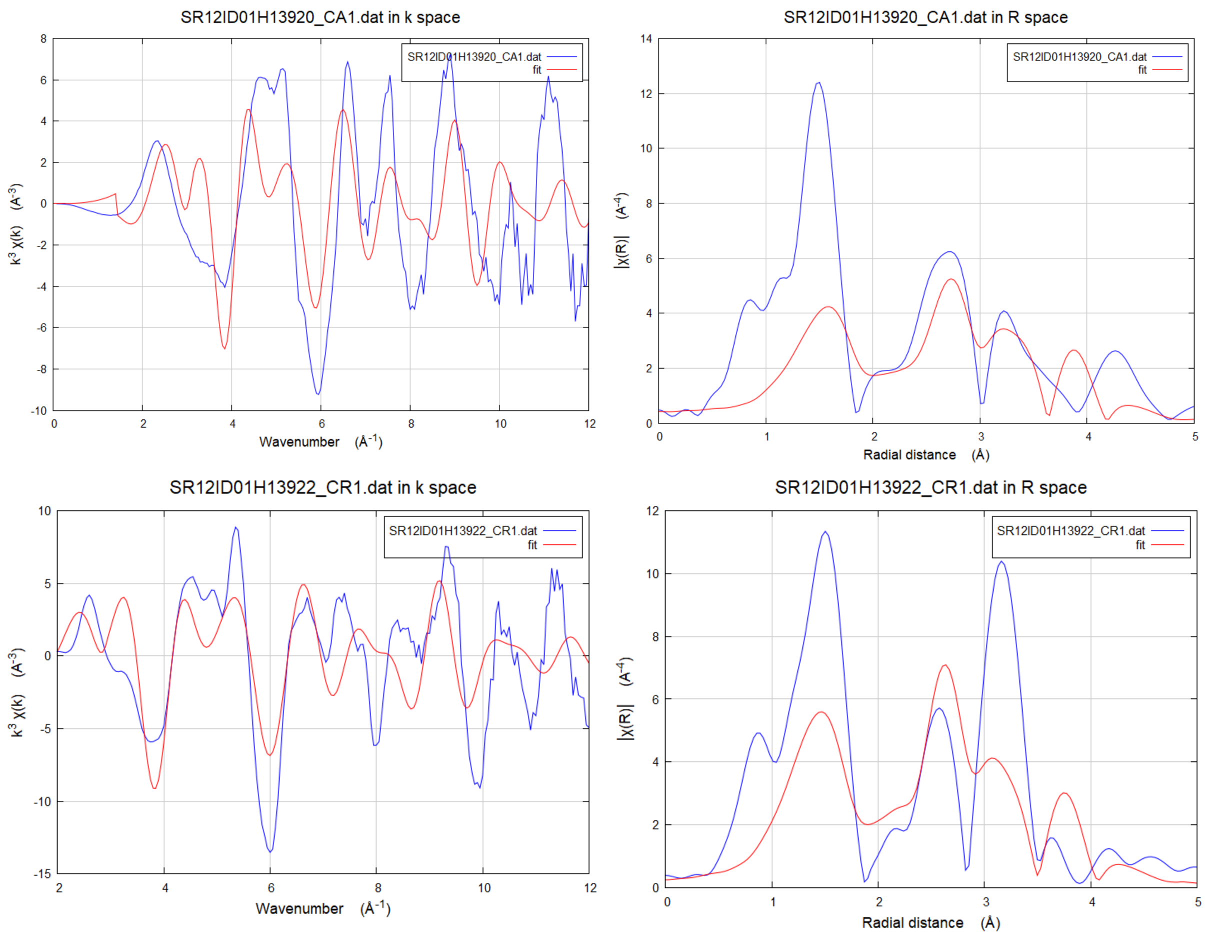

Figure S1. Fit (red line) to recorded data (blue line) of Nb-doped anatase (top) and Nb-doped rutile (bottom) to CIF 9008684 NbO F m -3 m

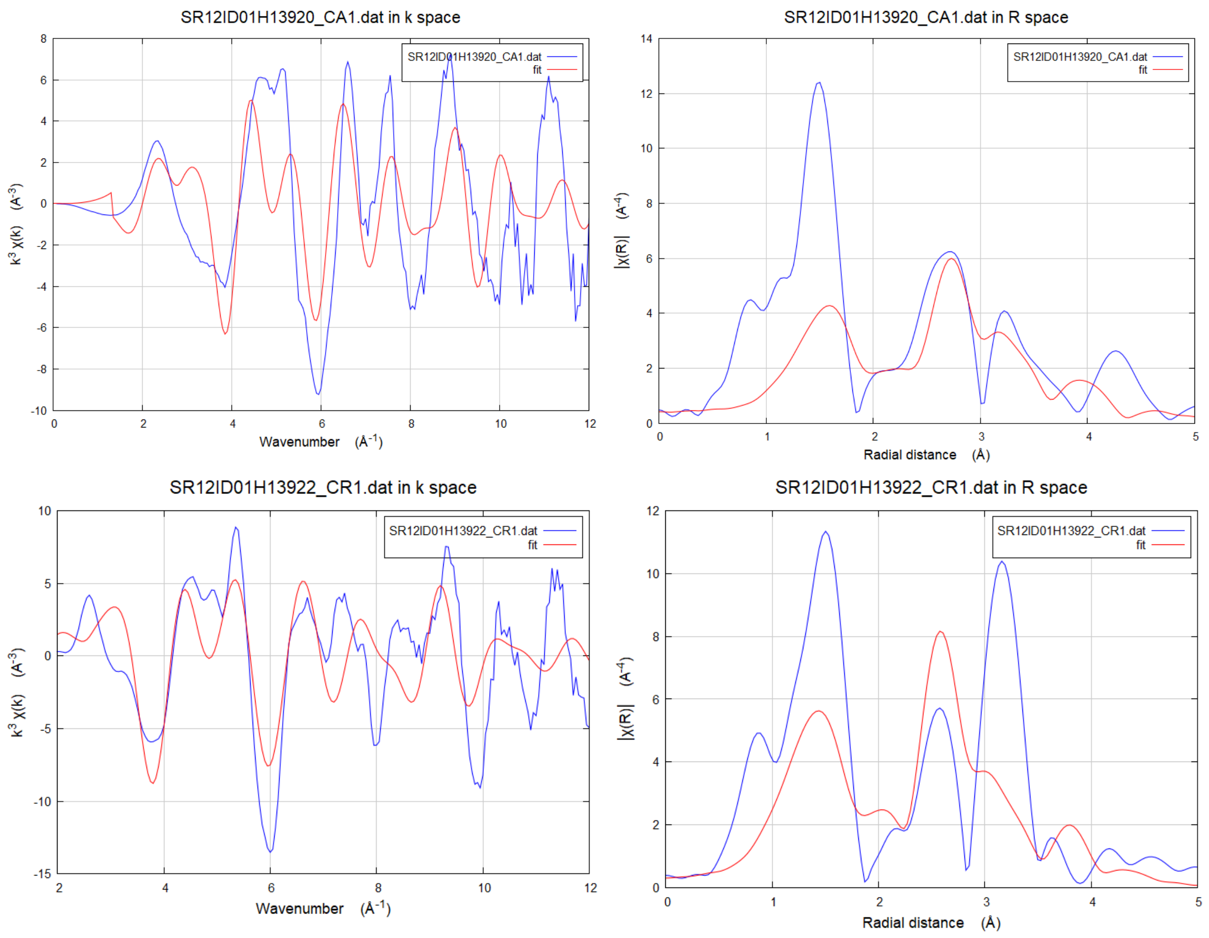

Figure S2. Fit (red line) to recorded data (blue line) of Nb-doped anatase (top) and Nb-doped rutile (bottom) to CIF 9008782 NbO P m -3 m

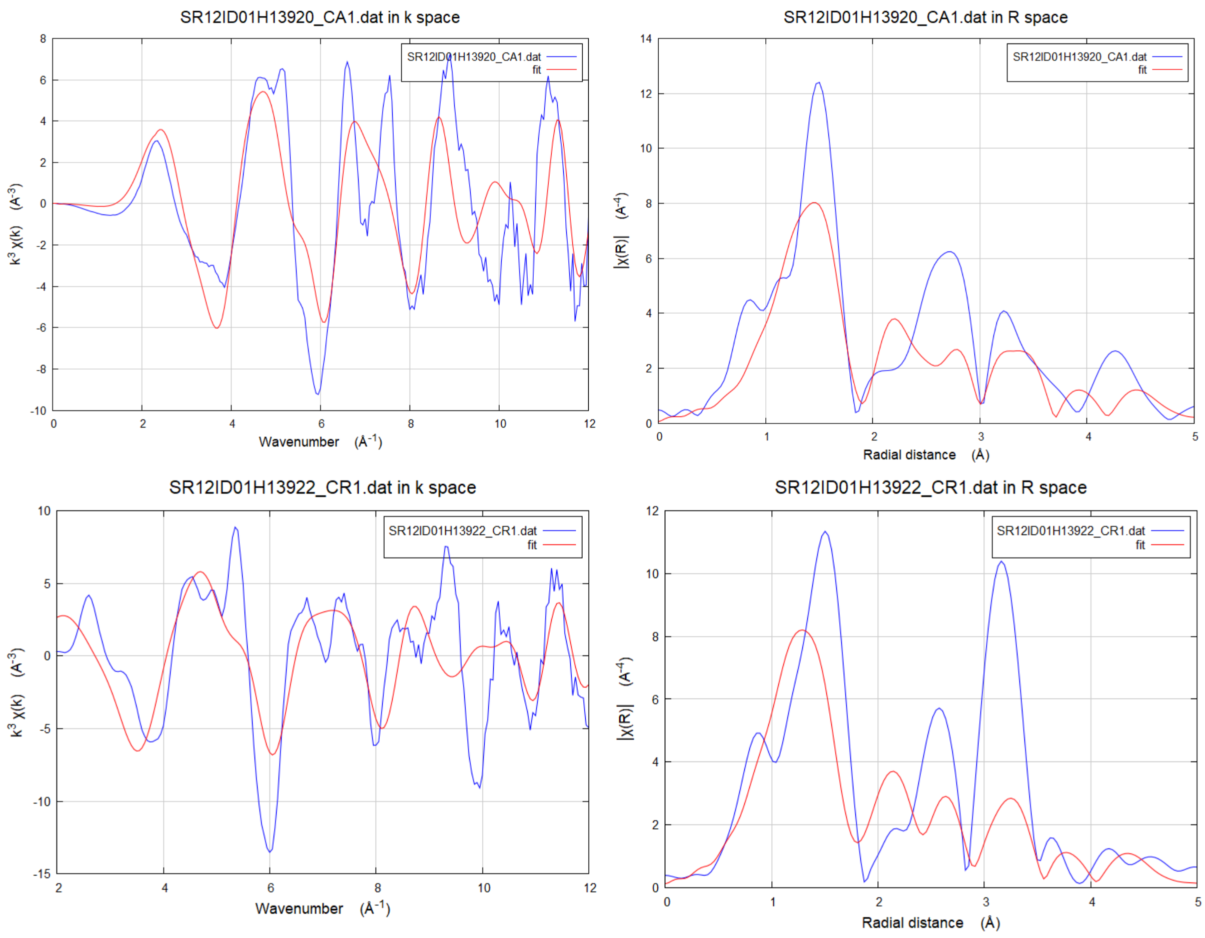

Figure S3. Fit (red line) to recorded data (blue line) of Nb-doped anatase (top) and Nb-doped rutile (bottom) to CIF 1520791 NbO_2 I 41/a :2

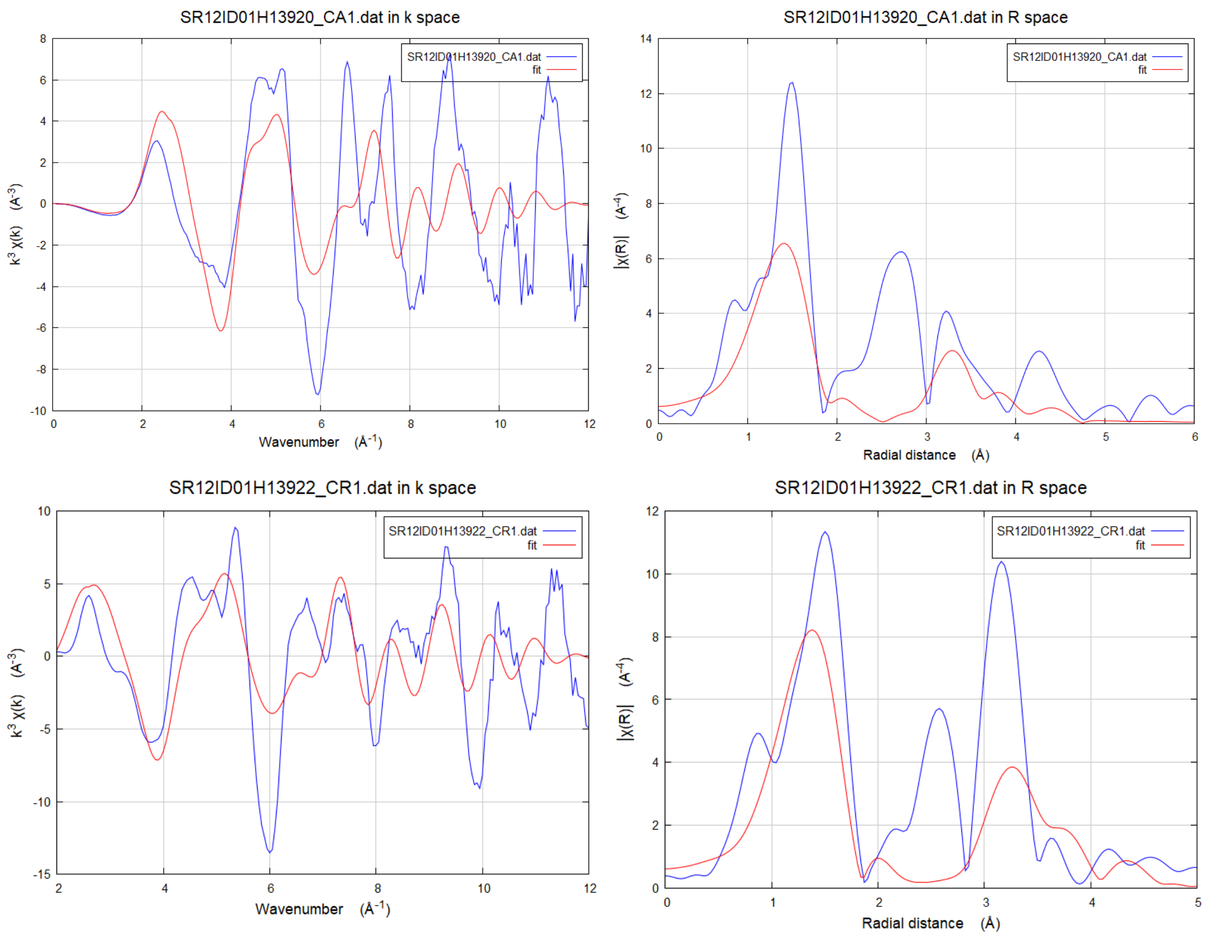

Figure S4. Fit (red line) to recorded data (blue line) of Nb-doped anatase (top) and Nb-doped rutile (bottom) to CIF 1534156 Nb_2O_5 C1 2/m 1

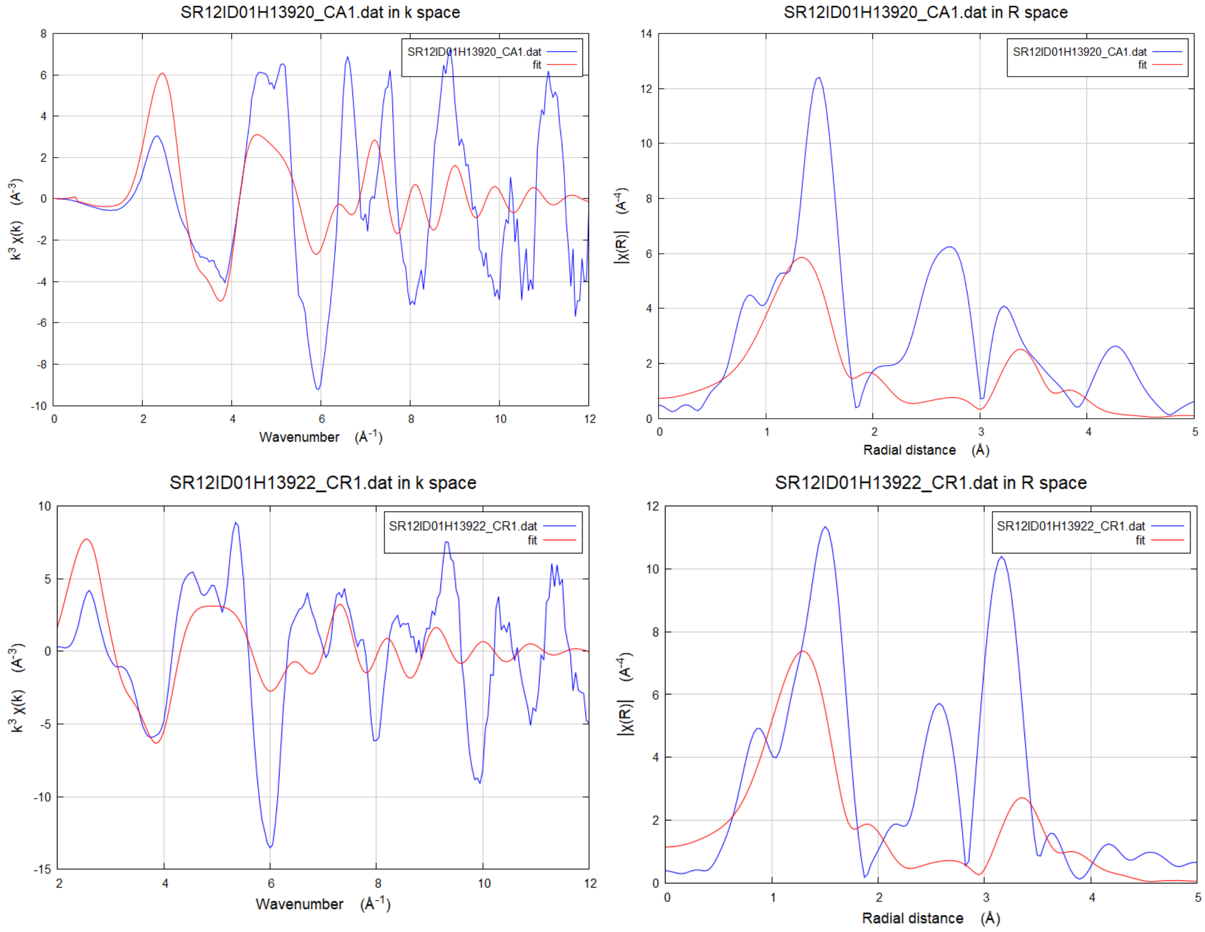

Figure S5. Fit (red line) to recorded data (blue line) of Nb-doped anatase (top) and Nb-doped rutile (bottom) to CIF 1528723 Nb_2O_5 I 4/m m m

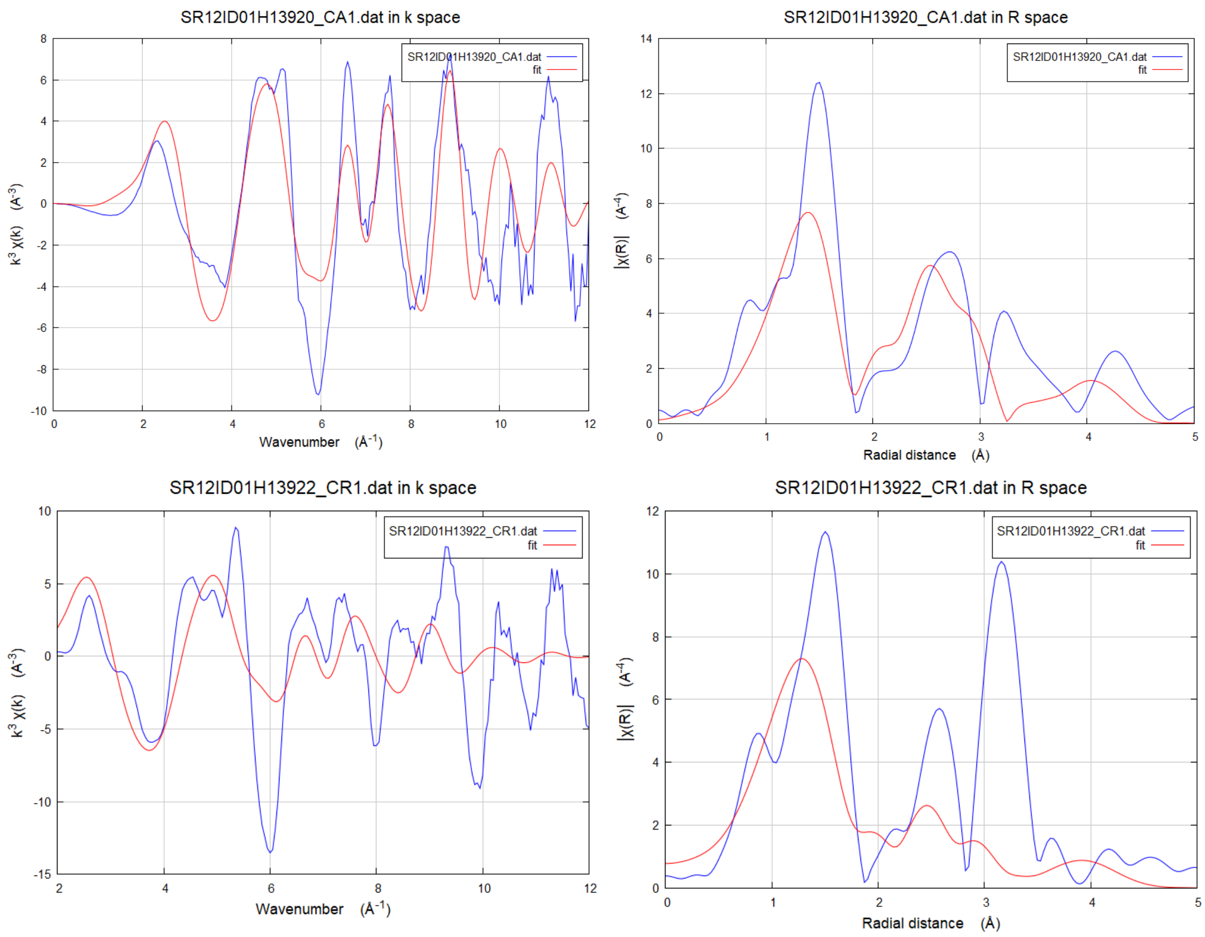

Figure S6. Fit (red line) to recorded data (blue line) of Nb-doped anatase (top) and Nb-doped rutile (bottom) to CIF 1534619 Nb_4O_5 P 42/n m c :2
